# Predicting COVID-19 cases with unknown homogeneous or heterogeneous resistance to infectivity

**DOI:** 10.1371/journal.pone.0254313

**Published:** 2021-07-15

**Authors:** Ramalingam Shanmugam, Gerald Ledlow, Karan P. Singh

**Affiliations:** 1 School of Health Administration, Texas State University, San Marcos, Texas, United States of America; 2 Department of Healthcare Policy, Economics and Management, School of Community and Rural Health, The University of Texas Health Sciences Center at Tyler, Tyler, Texas, United States of America; 3 Department of Epidemiology and Biostatistics, School of Community and Rural Health, The University of Texas Health Sciences Center at Tyler, Tyler, Texas, United States of America; University of Alabama at Birmingham, UNITED STATES

## Abstract

We present a restricted infection rate inverse binomial-based approach to better predict COVID-19 cases after a family gathering. The traditional inverse binomial (IB) model is inappropriate to match the reality of COVID-19, because the collected data contradicts the model’s requirement that variance should be larger than the expected value. Our version of an IB model is more appropriate, as it can accommodate all potential data scenarios in which the variance is smaller, equal, or larger than the mean. This is unlike the usual IB, which accommodates only the scenario in which the variance is more than the mean. Therefore, we propose a refined version of an IB model to be able to accommodate all potential data scenarios. The application of the approach is based on a restricted infectivity rate and methodology on COVID-19 data, which exhibit two clusters of infectivity. Cluster 1 has a smaller number of primary cases and exhibits larger variance than the expected cases with a negative correlation of 28%, implying that the number of secondary cases is lesser when the number of primary cases increases and vice versa. The traditional IB model is appropriate for Cluster 1. The probability of contracting COVID-19 is estimated to be 0.13 among the primary, but is 0.75 among the secondary in Cluster 1, with a wider gap. Cluster 2, with a larger number of primary cases, exhibits smaller variance than the expected cases with a correlation of 79%, implying that the number of primary and secondary cases do increase or decrease together. Cluster 2 disqualifies the traditional IB model and requires its refined version. The probability of contracting COVID-19 is estimated to be 0.74 among the primary, but is 0.72 among the secondary in Cluster 2, with a narrower gap. The advantages of the proposed approach include the model’s ability to estimate the community’s health system memory, as future policies might reduce COVID’s spread. In our approach, the current hazard level to be infected with COVID-19 and the odds of not contracting COVID-19 among the primary in comparison to the secondary groups are estimable and interpretable.

## Background and motivation

COVID-19 is the third-leading cause of death in 2020 in the USA, Belgium, France, Sweden, and the UK, behind only heart disease and cancer (www.kff.org). Predicting a pandemic like COVID-19 is challenging [1]. Major practical reasons that have been cited include poor data, insensitive parameter estimates, and imprecise epidemiologic features. A discussion of an underlying model for the data is missing from this list. What is a model? The model is an abstraction of reality; the better the model, the better it represents reality. The occurrence and spread of COVID-19 are too complex to be well matched by any known model in the literature. It is not surprising that the efforts to predict COVID-19 result in a failure, due to utilizing an unsuitable model for the data. To avoid failure, we must start by refining the model to suit the complexities that exist in the dynamic nature of COVID-19. That is exactly the research theme of this article. Of course, the main objective of any data collection is to predict future incidences as accurately as possible in order to be prepared for any emergencies and contingencies.

The professionals occasionally hear and/or argue that all models are wrong, but some are useful [2, 3]. Recently, in Shanmugam [4], a probabilistic approach is presented to capturing the impact of healthcare efforts on the prevalence rate of COVID-19’s infectivity, hospitalization, recovery, and mortality rates in the USA. Several non-intuitive findings including the existence of imbalance, different vulnerabilities, and risk reduction were noticed. In Shanmugam [5], the number of COVID-19 cases confirmed, recovered/cured, and fatalities across thirty-two of India’s states/territories, as of May 1, 2020, were modelled and analyzed. In the end, the attained administrative efficiency by the government was scrutinized. This knowledge leads to valuable lessons for adaptation for use in any future pandemics like COVID-19.

Hence, via modeling by trial and error, the professionals attempted to catch up and unravel the mysterious nature of the pandemic. This article is an attestation of such a hubristic endeavor to predict COVID-19 infectivity with a refined version of the traditional inverse binomial (IB) model. The traditional IB model possesses a unique property that the variance is larger than its expected value [6] and it limits its suitability for COVID-19 data under such variance. A refined version of the IB model is necessary to accommodate data under equal and over variance scenarios as they occur in COVID-19 data.

Yet, the issue of under variance seems not to have received enough attention compared to over variance in the literature [7, 8]. The Poisson model requires the equality of expected value and variance [6]. To deal with a deviation from such a requirement in Poisson data, in Conway [9], a mathematically complex approach is presented by raising a function of the observables to an unknown versatile parameter *τ*. Conway and Maxwell applied their approach to model the queuing systems with state-dependent service rates.

We now return to discuss a refined version of the IB model for COVID-19 data. At a fixed incidence rate, the number of COVID-19 cases is, of course, a Poisson type random variable. When COVID-19’s incidence rate is stochastically changing due to a variety of reasons as a gamma probability pattern, the convolution of Poisson and gamma probability structures results in the IB model for an occurrence of a random number of COVID-19 cases [10]. However, in this article, a refined and viable alternative to dealing with equal/under/over variance COVID-19 data, we start at its *jump start probability*. In Shanmugam [11], a concept of jump start probability in health/medical data analysis is introduced. The method in this article is simple, easy, and versatile, but originates from the jump probability. The unsteady nature of the variance is recognized here as the *heterogeneity* level in the gatherings of family with some members being carriers of the COVID-19 virus. Heterogeneity causes the prediction of COVID-19 incidence rates to be haphazard, if not uncanny.

COVID-19 was first noticed in Wuhan Province in the central part of China. Those with COVID-19 symptoms leaving Wuhan to participate in family gatherings in Gansu Province are recognized as the primary cases [12]. Those at the family gatherings in Gansu Province who were infected by the primary cases are recognized as secondary cases. Is there a significant difference in the virality among the secondary cases in comparison to the primary cases? This research question is answered in the second part of this article. For this purpose, new concepts of infectivity that restricted IB modeling processes are defined, and they are utilized to derive analytic expressions with an intention to better predict the future COVID-19 infection rates and cases. We illustrate our methodology using the COVID-19 data given in Fan [12].

## Uneven COVID-19 jump start proportion

To ease the comprehension of the derivation of an infectivity rate restricted version of the inverse binomial (IB) which adds on under and equal variance properties in addition to preserving already existing over variance properties, we start with the COVID-19 scenarios. Suppose there is an unknown risk level, 0 < *θ* < 1 for a person to contract COVID-19 at a family gathering. Also, assume that there exists an unknown restriction level, *τ* > 1 on infectivity due to persons who may have a strong immunity and/or have undertaken strict preventive measures such as physical social distancing, wearing face coverings, washing hands with soap/sanitizer frequently, etc. Consequently, the original risk level, *θ* changes to a new risk level $\frac{\theta }{{\left(1-\theta \right)}^{\left(\tau -1\right)}}$, where *τ* > 1. Here, the non-negative parameter *τ* is to indicate the heterogeneous resistance level to COVID-19’s infectivity potential.

When *τ* > 1, it is indicative of heterogeneously resistant to the COVID-19’s virus among the participants. Notice that the new risk level validates the requirement that $0<\frac{\theta }{{\left(1-\theta \right)}^{\left(\tau -1\right)}}<1$, if $\tau >1+\frac{\text{ln}\;\theta }{\text{ln}(1-\theta )}$. The expression $0<\frac{\theta }{{\left(1-\theta \right)}^{\left(\tau -1\right)}}<1$ is valid provided the condition is true. In other words, there is an interplay between 0 < *θ* < 1 and *τ* > 1 as they shift in their domains. In this “game-theoretic” operations, when *θ* shifts in its domain (0,1), the companion parameter *τ* alters its domain according to the derived restriction.

**Fig 1:**
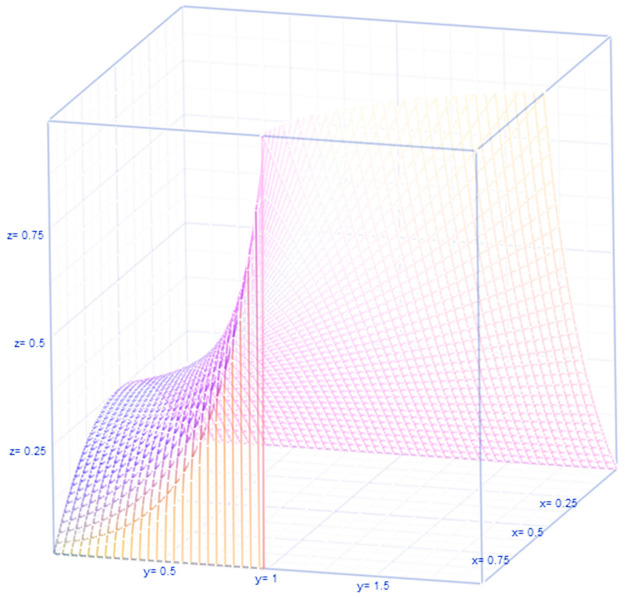
Interplay between *θ* and *τ* for $0<\frac{\theta }{{\left(1-\theta \right)}^{\left(\tau -1\right)}}<1$.

See Fig 1 giving the parameter constraint and illustrating the interplay between *θ* and *τ* for $0<\frac{\theta }{{\left(1-\theta \right)}^{\left(\tau -1\right)}}<1$.

Hence, it results in a restricted infection rate: $0<\theta <\frac{1}{\tau }<1$ with *τ* > 1. In the case of *τ* > 1, any participant’s chance of being safe from contracting COVID-19 is $0<1-\frac{\theta }{{\left(1-\theta \right)}^{\left(\tau -1\right)}}<1$ after the family gathering, while her/his chance of contracting COVID-19 is $0<\frac{\theta }{{\left(1-\theta \right)}^{\left(\tau -1\right)}}<1$ after, as it happened in Gansu Province, China. Various above-mentioned preventive measures might not offer absolute safeguards from the COVID-19 infection potential all the time. Consequently, the odds of not contracting COVID-19 exist whether the participants are homogeneously immune (that is, *τ* = 1) or heterogeneously resistant (that is, *τ* > 1) to contract the COVID-19 virus. Hence, the odds for being safe after the family gathering is $\left\{\frac{{\left(1-\theta \right)}^{\left(\tau -1\right)}}{\theta }-1\right\}$ among the heterogeneously resistant participants as they identified by the infection rate restricted inverse binomial (IRRIB) model.

In the case of *τ* = 1, as the infectivity is *unrestricted* in a sense: 0 < *θ* < 1, any participant’s chance of being *safe* from contracting COVID-19 is 0 < 1 − *θ* < 1 after the family gathering, while her/his chance of contracting COVID-19 is 0 < *θ* < 1 after the family gathering as it happened in Gansu Province, China. Hence, the odds for being safe after the family gathering is $\frac{\left(1-\theta \right)}{\theta }$ among the homogeneously immune participants as they identified by the IB model. The traditional IB model is an extremely special case of the IRRIB model when *τ* = 1, because the denominator (1 − *θ*)^(*τ*−1)^ = 1 as a baseline value. In other words, when the family gathering consists of attendees having homogeneous immunity to COVID-19 (that is, *τ* = 1), using the traditional IB model is meaningful. Note that gathering is defined as an episode where three or more family members are visiting together in the same social space for more than 15 minutes.

Otherwise (that is, with a family gathering in which the attendees have heterogeneous resistance to COVID-19), the involvement of *τ* > 1 is a necessity in modeling to predict future COVID-19 cases. Hence, the modeling approach based on IRRIB is versatile to predict incidence of COVID-19 in all three (equal, under, and over variance) scenarios mentioned above.

We now discuss the odds of not contracting COVID-19 among attendees having homogeneous resistance to COVID-19 versus heterogeneous resistance to COVID-19 in the gathering. Amid homogeneous resistance to COVID-19 family members in the gathering, the factor $\frac{\left(1-\theta \right)}{\theta }$ is recognized as the odds for not contracting COVID-19, and it is a safe situation. The odds for a safe situation in the IRRIB process (synonymous to a situation in which the family gathering involves attendees having heterogeneous resistance to COVID-19) against contracting COVID-19 is

$$Odds_{\tau \neq 1}^{safe\;}=\frac{\text{Pr}(Y=0)}{\text{Pr}(Y\geq 1)}={\left[{\left\{1-\frac{\theta_{\tau \neq 1}}{{\left(1-\theta_{\tau \neq 1}\right)}^{\left(\tau -1\right)}}\right\}}^{-m\tau }-1\right]}^{-1};0<\theta <\frac{1}{\tau }<1$$ (1)

in comparison to the $Odds_{\tau =1}^{safe}={\left[{\left(1-\theta_{\tau =1}\right)}^{-m}-1\right]}^{-1};0<\theta <1$ for safe in the IB process (synonymous to a situation in which the family gathering involves attendees having homogeneous resistance to COVID-19). The odds ratio in family gatherings in which the attendees having heterogeneous resistance to COVID-19 against having homogeneous resistance to COVID-19 is

$$OR_{\frac{\tau \neq 1}{\tau =1}}=\frac{Odds_{\tau \neq 1}}{Odds_{\tau =1}}=\frac{\left[{\left(1-\theta_{\tau =1}\right)}^{-m}-1\right]}{\left[{\left\{1-\frac{\theta_{\tau \neq 1}}{{\left(1-\theta_{\tau \neq 1}\right)}^{\left(\tau -1\right)}}\right\}}^{-m\tau }-1\right]};0<\theta <\frac{1}{\tau }<1,$$ (2)

The “m” is called “convolution parameter” [13]. It means that if *Y*_1_ with *m*_1_ > 0 and *Y*_2_ with *m*_2_ > 0 are two independent data realizations/sources of an inverse binomial population with same *θ*, then their convolution *Y* = *Y*_1_ + *Y*_2_ will follow an inverse binomial probability pattern with *m* = *m*_1_ + *m*_2_ > 0. This concept of “convolution” paves the way for the concept of “infinite divisibility” in statistical inference. For an example of it, when *m* = 1, the inverse binomial reduces to a geometric sampling situation.

Note that Eq (1) is the “odds of being safe under heterogeneous” situation. Eq (2) is the odds ratio of the odds of being safe under heterogenous situation over the odds of being safe under homogenous situation. As mentioned before, we do the following in this article. There are two situations, which we do not know because of ungiven information. This refers to a situation in which the “hosts” and “guests” are homogenous with respect to “COVID-19 infectivity” and refers to a situation in which the “hosts” and “guests” are heterogenous with respect to “COVID-19 infectivity.” For example, if a person in the gathering is already “vaccinated” and another in the gathering is “not vaccinated”, they are heterogeneous. Data are not providing information about the heterogeneity or homogeneity. Our model is an approach to contemplate how the outcome might be different under heterogeneous from that of homogeneous situations.

## Jump rate-incidence of COVID-19 cases

We now move on to discuss the jump rate in the incidence of COVID-19 cases. Furthermore, let us assume that there are *m* ≥ 1 family gatherings with a group of participants with heterogeneous resistance to COVID-19, *τ* ≥ 1 at the family gathering. Let *Y* be a random number of COVID-19 cases emerging out of the *m* family gatherings. Notice that the probability for a single new COVID-19 case to arise in any gathering is $\text{Pr}(Y=1)=\frac{\theta }{{\left(1-\theta \right)}^{\left(\tau -1\right)}}$ in comparison to the probability of an infection-free situation, $\text{Pr}(Y=0)=1-\frac{\theta }{{\left(1-\theta \right)}^{\left(\tau -1\right)}}$.

The jump rate detailed in [11] from a COVID-19 free situation to the pandemic is given by:

$$jumpRate_{\left(\theta ,\tau \right)}=\frac{\text{Pr}(Y=1)}{\text{Pr}(Y=0)}={\left(\frac{{\left(1-\theta \right)}^{\left(\tau -1\right)}}{\theta }-1\right)}^{-1}$$ (3)

if the family gatherings involve attendees having heterogeneous resistance to COVID-19. When the gathering consists of attendees having homogeneously immunity to COVID-19 (i.e., *τ* = 1), the jump rate from COVID-19 free status to contracting COVID-19 is simply $jumpRate_{\left(\theta ,\tau =1\right)}=\frac{\theta }{\left(1-\theta \right)}$ and it pertains to the ideal, traditional IB model scenario for any communicable disease but not necessarily the highly infectious and treacherous COVID-19 scenario. Now, consider a domino effect of this jump rate, especially in the COVID-19 situation. Their recursive probabilities are connected to the jump rate as follows.


$$\text{Pr}(Y=y)=\left(\frac{m\tau +y-1}{y}\right)\left\{\frac{\theta }{{\left(1-\theta \right)}^{\left(\tau -1\right)}}\right\}\text{Pr}(Y=y-1);y=0,1,2,\ldots ;0<\theta <\frac{1}{\tau }<1.$$ (4)

**Fig 2:**
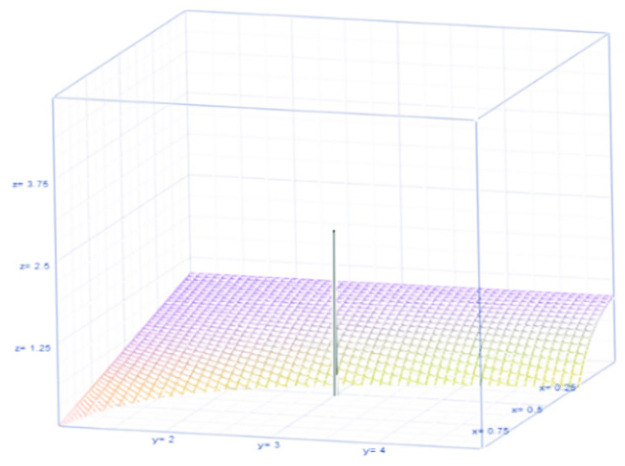
Probability of being safe.

That is,

$$\text{Pr}(Y=y)\propto \frac{\Gamma (m\tau +y)}{y!\Gamma (m\tau )}{\left\{\frac{jumpRate{\;}_{\left(\theta ,\tau \right)}}{1+jumpRate_{\left(\theta ,\tau \right)}}\right\}}^{y};y=0,1,2,\ldots ;0<\theta <\frac{1}{\tau }<1$$ (5)

with an appropriate normalizer

$$N(\theta ,\tau )={\left\{1-\frac{\theta }{{\left(1-\theta \right)}^{\left(\tau -1\right)}}\right\}}^{m\tau }={\left\{1+jumpRate{\;}_{\left(\theta ,\tau \right)}\right\}}^{-m\tau }$$ (6)

(its dynamic is illustrated in Fig 2). The dynamic nature of

$$\frac{{\left(1-\theta \right)}^{\left(\tau -1\right)}}{\theta }=1+\frac{1}{jumpRate_{\left(\theta ,\tau \right)}}$$ (7)

is noticed in Fig 1. By imposing *τ* = 1 or *m* = 1, the traditional IB model which is often employed to deal with a group homogeneously immune to COVID-19 after the family gathering.

On the contrary, together when *mτ* = 1, they reduce to what we wish to call the infection rate restricted geometric (IRRG) process. That is,

$$\text{Pr}(Y=y|m\tau =1)=\left\{1-\frac{\theta }{{\left(1-\theta \right)}^{\left(\tau -1\right)}}\right\}\frac{1}{y!}{\left\{\frac{\theta }{{\left(1-\theta \right)}^{\left(\tau -1\right)}}\right\}}^{y};y=0,1,2,\ldots ;0<\theta <\frac{1}{\tau }<1.$$ (8)

For probability of *safe*, $\left\{1-\frac{\theta }{{\left(1-\theta \right)}^{\left(\tau -1\right)}}\right\}$, see Fig 2.

**Fig 3:**
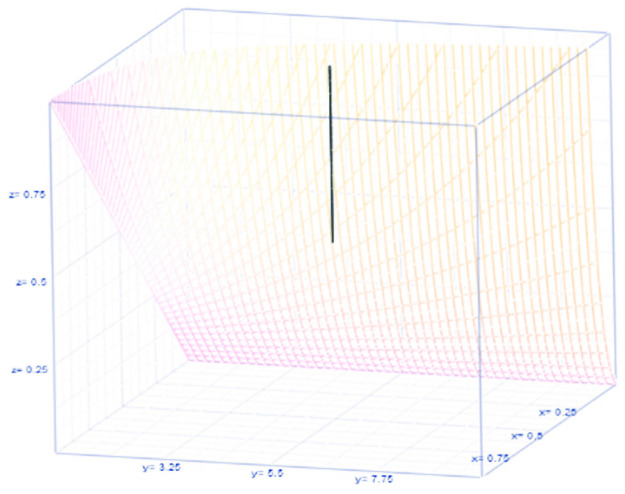
Risk for having COVID-19.

For risk of COVID-19, $\frac{\theta }{{\left(1-\theta \right)}^{\left(\tau -1\right)}}$, see Fig 3.

However, it is easy to notice that the expected value, *E*(*Y*) and variance, *Var*(*Y*) of the IRRIB model are, respectively,

$$E(Y)=\overset{\infty }{\underset{y=0}{\sum }}yN(\theta ,\tau )\text{Pr}(Y=y)=m\tau \left\{1+\frac{1}{jumpRate_{\left(\theta ,t\right)}}\right\}$$ (9)

And

$$\text{Var}(Y)=\left\{1+\frac{1}{\;jumpRate{\;}_{\left(\theta ,\tau \right)}}\right\}E(Y).$$ (10)

Mathematical derivations with explanations for the sake of predicting future COVID-19 cases are given in S1 Appendix.

## Prediction of COVID-19 cases after family gatherings

To illustrate the main concepts and analytic expressions in Section 2, let us consider the COVID-19 data connecting Gansu Province (32°31’N–42°57’N, 92°13’E–108°46’E), in Northwest China and Wuhan (30.5928° N, 114.3055° E) in Central China. COVID-19 was first noticed on December 31, 2019, in Wuhan, China. Wuhan is connected to Gansu by travel options including airplanes, railroads, interstate buses, and private cars. The COVID-19 virus originated in Wuhan.

The primary and secondary COVID-19 cases are defined as follows. The primary cases refer to those who traveled from Wuhan to Gansu. The secondary COVID-19 cases refer those who never left Gansu. The secondary COVID-19 cases were the outcome of family gatherings in which the primary and secondary COVID-19 cases mingled together. In other words, the secondary cases might have been infected by the primary cases. It is assumed in [12] that the COVID-19 virus does not mutate to reduce its virulence in transmission. One then wonders whether the secondary cases exhibit different characteristics from that of primary cases. This question is the research aim of this article.

**Fig 4:**
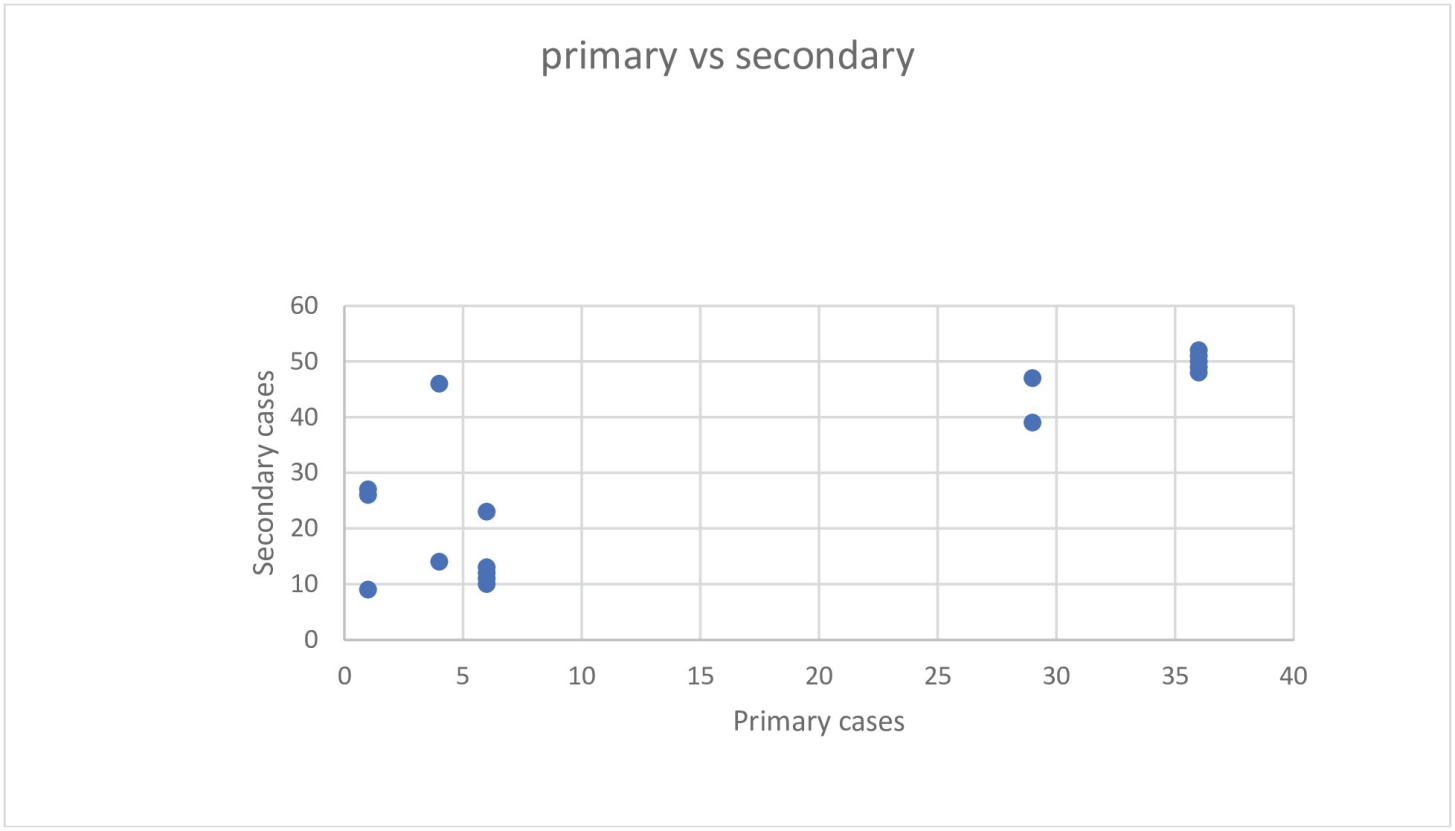
Primary versus secondary cases of COVID-19.

We want to capture and compare the virality of the COVID-19 incidence rates/cases in the primary versus secondary groups. When we plot them in the same graph, we noticed that there are two clusters in the data (see Fig 4). Table 1 (with gatherings of ten families) contains lesser primary cases, while Table 2 (with gatherings of seven families) contains larger primary cases of COVID-19. See Fig 4 for primary vs. secondary COVID-19 cases.

**Table 1:** The family gathering in which the primary cases were in single digit (with over variance).

Family Gathering	*Y*_*p*_ = Primary COVID-19 Cases	*Y*_*s*_ = Secondary COVID-19 Cases
1	1	9
2	1	26
3	1	27
4	4	14
5	4	46
6	6	10
7	6	11
8	6	12
9	6	13
n = 10	6	23
$\bar{y}=\sum_{i=1}^{n}y_{i}/n$	4.1	19.1
$s_{y}^{2}=\sum_{i=1}^{n}{\left(y_{i}-\tilde{y}\right)}^{2}/(n-1)$	5.21 Over Variance	134.76 Over Variance
*m* = # unions	41	191
Correlation (*Y*_*p*_, *Y*_*s*_)	-0.28	
${\hat{\tau }}_{mle}$ (if homogeneously immune to COVID-19)	1	1
${\hat{\theta }}_{mle}$ (if homogeneously immune to COVID-19)	0.78	0.14
${\hat{\tau }}_{mle}$ (if *heterogeneously resistance to COVID-19*)	2.27	8.05
${\hat{\theta }}_{mle}$ (if *heterogeneously resistance to COVID-19*)	0.55	0.87
$Odds_{\tau =1}^{safe\;}$ (if *homogeneously immune to COVID-19)*	0.27	6.05
$Odds_{\tau \neq 1}^{safe\;}$ (if *heterogeneously resistance to COVID-19)*	0.37	0.99
ORτ≠1τ=1	1.36	0.16
$E\left\{\frac{{\bar{y}}^{2}}{\left|s_{y}^{2}-\bar{y}\right|}|H_{o}\right\}$ (if *homogeneously immune to COVID-19)*	4.56	19.11
$\sqrt{Var\left\{\frac{{\bar{y}}^{2}}{\left|s_{y}^{2}-\bar{y}\right|}|H_{o}\right\}}$ (if *homogeneously immune to COVID-19*)	3.59	2.71
$E\left\{\frac{{\bar{y}}^{2}}{\left|s_{y}^{2}-\bar{y}\right|}|H_{1}\right\}$ (family gathering consists of attendees having *heterogeneous resistance to COVID-19*)	10.29	149.54
$\sqrt{Var\left\{\frac{{\bar{y}}^{2}}{\left|s_{y}^{2}-\bar{y}\right|}|H_{1}\right\}}$ (family gathering consists of attendees having *heterogeneous resistance to COVID-19*)	13.09	1081.33
Test score $\frac{{\bar{y}}^{2}}{\left|s_{y}^{2}-\bar{y}\right|}$	15.12	3.15
p Value	0.99	1.85043E-22
Power	0.76	0.99

**Table 2:** The family gathering in which the primary cases were in double digits (with under variance).

Family Gathering	*Y*_*p*_ = Primary COVID-19 Cases	*Y*_*s*_ = Secondary COVID-19 Cases
11	29	39
12	29	47
13	36	48
14	36	49
15	36	50
16	36	51
17 (n = 7)	36	52
$\bar{y}=\sum_{i=1}^{n}y_{i}/n$	34	48
$s_{y}^{2}=\sum_{i=1}^{n}{\left(y_{i}-\tilde{y}\right)}^{2}/(n-1)$	11.66 Under Variance	18.66 Under Variance
*m* = # family gatherings	238	336
Corr (*Y*_*p*_, *Y*_*s*_)	0.79	079
${\hat{\tau }}_{mle}$ (if homogeneously immune to COVID-19)	1	1
${\hat{\theta }}_{mle}$ (if homogeneously immune to COVID-19)	0.25	0.28
${\hat{\tau }}_{mle}$ (if *heterogeneously resistance to COVID-19*)	1.34	1.38
${\hat{\theta }}_{mle}$ (if *heterogeneously resistance to COVID-19*)	0.25	0.28
$Odds_{\tau =1}^{safe\;}$ (if *homogeneously immune to COVID-19)*	1.34	1.38
$Odds_{\tau \neq 1}^{safe\;}$ (if *heterogeneously resistance to COVID-19)*	2.91	2.57
ORτ≠1τ=1	2.53	2.14
$E\left\{\frac{{\bar{y}}^{2}}{\left|s_{y}^{2}-\bar{y}\right|}|H_{o}\right\}$ (if *homogeneously immune to COVID-19)*	0.87	0.83
$\sqrt{Var\left\{\frac{{\bar{y}}^{2}}{\left|s_{y}^{2}-\bar{y}\right|}|H_{o}\right\}}$ (if *homogeneously immune to COVID-19*)	39.69	56.02
$E\left\{\frac{{\bar{y}}^{2}}{\left|s_{y}^{2}-\bar{y}\right|}|H_{1}\right\}$ (family gathering consists of attendees having *heterogeneous resistance to COVID-19*)	10.15	15.70
$\sqrt{Var\left\{\frac{{\bar{y}}^{2}}{\left|s_{y}^{2}-\bar{y}\right|}|H_{1}\right\}}$ (family gathering consists of attendees having *heterogeneous resistance to COVID-19*)	53.30	77.80
Test score $\frac{{\bar{y}}^{2}}{\left|s_{y}^{2}-\bar{y}\right|}$	15.08	24.77
p Value	0.01	0.01
Power	0.98	0.99

Comparing the sample expected value and variance of the primary and secondary cases given in Tables 1 and 2, there exists over (under) variance. Fig 1 is to notice that there are two clusters in the union data. The over (under) variance is synonymous with the null hypothesis statement *H*_0_: *τ* = 1 (with the alternative statement *H*_1_: *τ* ≠ 1). For both the data, the test score, $\frac{{\bar{y}}^{2}}{\left|s_{y}^{2}-\bar{y}\right|}$ was computed and displayed in Tables 1 and 2. Utilizing all the derived expressions, the values of ${\hat{\theta }}_{mle,H_{o}}$ under

$$H_{0}:\tau =1,E\left\{\frac{{\bar{y}}^{2}}{\left|s_{y}^{2}-\bar{y}\right|}|H_{o}\right\},\sqrt{Var\left\{\frac{{\bar{y}}^{2}}{\left|s_{y}^{2}-\bar{y}\right|}|H_{0}\right\}},$$ (11)

correlations under *H*_0_: *τ* = 1 in a situation in which the family gathering consists of attendees having heterogeneous resistance to COVID-19 are calculated and displayed in Tables 1 and 2. The values of ${\hat{\tau }}_{mle,H_{1}}$ under *H*_1_: *τ* ≠ 1, ${\hat{\theta }}_{mle,H_{1}}$ under

$$H_{1}:\tau \neq 1,E\left\{\frac{{\bar{y}}^{2}}{\left|s_{y}^{2}-\bar{y}\right|}|H_{1}\right\},\sqrt{Var\left\{\frac{{\bar{y}}^{2}}{\left|s_{y}^{2}-\bar{y}\right|}|H_{1}\right\}},$$ (12)

and correlations under *H*_1_: *τ* ≠ 1 in a situation in which the family gathering consists of attendees having heterogeneous resistance to COVID-19 are computed and displayed in Tables 1 and 2. The p-value and the statistical power are calculated and displayed in Tables 1 and 2. A comparison of them reveals that the p-values are significant for the primary cases and secondary cases in Tables 1 and 2. With a selection of $\tau ={\hat{\tau }}_{1}$ under the alternative hypothesis, the statistical power is calculated and displayed in Tables 1 and 2. The power is excellent for the primary and secondary cases in Tables 1 and 2. Next, we express final comments and conclusions with respect to predicting implications of our model and analytic results for this study concerning COVID-19.

### Cluster 1: Small primary cases

Here, the number of primary COVID-19 cases is in a single digit (lesser) as in Table 1. In this case, the number of gatherings is also less, the data have *over* variance than the expected value in both primary as well as secondary cases, the estimate of contracting COVID-19 in primary and secondary type is 0.78 and 0.14, respectively. With an estimate of the parameters in our mode, the prediction of a future number of primary and secondary COVID-19 cases will be 5 and 20, respectively, when the family gathering consists of attendees having *homogeneous resistance to COVID-19 and* having *heterogeneous resistance to COVID-19*, *respectively*, *in Cluster 1*. The p-value for the suitability of the IB process for COVID-19 data in Table 1 are 0.0001 in the case of primary as well as secondary infection. The *odds* for not contracting COVID-19 are 0.27 and 6.053 as primary and secondary, respectively, if the attendees are homogeneously immune to the COVID-19 virus. The estimate of contracting COVID-19 (in family gatherings in which the attendees have heterogeneous resistance to COVID-19) is 0.37 among primary cases and 0.99 among secondary cases. The (statistical) power of accepting the research hypothesis $H_{1}:\tau ={\hat{\tau }}_{mle}$ is 0.76 for the primary and 0.99 for the secondary when ${\hat{\tau }}_{mle}$ is the true value.

### Cluster 2: Large primary cases

Here, the number of primary COVID-19 cases is in double digits (larger), as in Table 2. In this case, the number of gatherings is also large, but the data have under variance than the expected value in both primary as well as secondary cases. The risk of contracting the COVID-19 virus in primary and secondary type is 0.25 and 0.28, respectively. With the estimate of the parameters in our model, the prediction of a future number of primary and secondary COVID-19 cases will be 40 and 56, respectively, when the family gathering consists of attendees having *homogeneous resistance to COVID-19 and* having *heterogeneous resistance to COVID-19*, *respectively*, *in Cluster 2*. The odds of not contracting COVID-19 in primary and secondary type are 2.53 and 2.143, respectively; the prediction of a future number of primary and secondary COVID-19 cases will be 40 and 56, respectively, when the family gathering consists of attendees having homogeneous resistance to COVID-19. The prediction of a future number of primary and secondary COVID-19 cases will be 54 and 78, respectively, when the family gathering consists of attendees having homogeneous resistance to COVID-19. The p-value for the suitability of IB model processes for the COVID-19 data in Table 2 are 0.006 in the case of primary and 0.0000058 in the case of secondary infection.

The odds of not contracting COVID-19 are 2.91 and 2.57 among the primary and secondary cases, respectively if the family gathering consists of attendees having homogeneous resistance to COVID-19. The odds for not contracting COVID-19 are 2.53 and 2.14 among the primary and secondary cases, respectively, if the family gathering consists of attendees having heterogeneous resistance to COVID-19. The estimate of not contracting COVID-19 (in family gatherings in which the attendees have heterogeneous resistance to COVID-19) is 0.984 among primary cases and 0.72 among secondary cases. The (statistical) power of accepting the research hypothesis $H_{1}:\tau ={\hat{\tau }}_{mle}$ are 0.984 for the primary cases and 0.998 for the secondary cases when ${\hat{\tau }}_{mle}$ is the true value.

## Discussion and concluding remarks

The COVID-19 pandemic is a challenge not only to healthcare professionals, policy makers, epidemiologists and biostatisticians who try to model data and make successful predictions, but also to the general public who faithfully adhere to recommendations such as social distancing, face covering, and utilizing good and frequent sanitizing practices. Despite all these precautionary efforts, many families desire to get together for occasions and events. Some participants in such gatherings may have originated from a place like Wuhan, China in where the COVID-19 virus was spreading, and they are labelled as primary cases (*Y*_*p*_).

The other participants at a gathering occurring at a location like Gansu Province, China where the COVID-19 virus had not yet appeared could get infected by the primary cases from Wuhan, and they are labelled as secondary cases (*Y*_*s*_) in the epidemiologic data collection process. An unknown in such gatherings is whether the participants are homogeneously immune to the infectivity or heterogeneously risky to contract the virus. Amid less clarity, an issue of interest might be how best one can predict the number of COVID-19 cases after the family gathering. In other words, a research goal for the analysts of infectious diseases is to address the similarities versus differences between the primary and secondary groups in the family gathering. The research goal appears simple and easy on the surface but is actually very complicated and challenging, as pointed out in a recent article [1].

We learned the following from the statistical analysis of COVID-19 data in Table 1 in this article. The spreading of the COVID-19 virus is expedited by family gatherings with attendees having heterogeneous resistance to COVID-19; the change is significantly different from its earlier risk of contracting COVID-19. In a macro sense, the number of secondary COVID-19 cases would have been much less if the number of primary COVID-19 cases was smaller in the first place. When these family gatherings contribute to massive infection incidents, many communities, not to mention entire nations, do not have enough resources to treat a deluge of patients. When citizens are locked down in homes without working, the nations’ productivity reduces to near zero levels, and the global economy suffers consequently. The absence of vaccinations to prevent the spread of COVID-19 makes the scenario bleaker, although several countries have faithfully committed to social distancing, wearing face coverings. and other mitigation regimens. It seems that we all have a long way to go to reach the day in which the virus of COVID-19 is totally controlled and eventually eradicated. To attain this optimum level, professionals need to do more research work with pertinent data on COVID-19.

The advantages of this new approach include the model’s ability to estimate the community’s health system memory for future policy development, as such policies might reduce the COVID-19 viral spread in an effort to control the pandemic. In our approach, as demonstrated, the current hazard level to become infected with COVID-19 and the odds of not contracting COVID-19 among the primary in comparison to the secondary groups are estimable and interpretable. In essence, family gatherings, especially with more vulnerable family members who are aged, have chronic diseases, or have issues of reduced immunity, need to be highly scrutinized by the family members before engaging in such events. Computer mediated communication such as tele/video conferencing should be “pushed” and possibly subsidized by each governmental level to encourage family interaction but while utilizing safe venues. What was omitted in the itemized reasons in [1] for not successfully predicting COVID-19 cases is the role of an appropriate underlying model for the data. A model is an abstraction of reality. To rectify this situation, this article has constructed and illustrated a restricted infection rate inverse binomial-based approach to better predict future COVID-19 cases after a family gathering or social event.

## Supporting information

S1 Text *Emerging infectious diseases* June 2020: 26 (6): 1257–1265. https://wwwnc.cdc.gov/eid.
(TXT)

S1 Appendix (DOCX)
