# Supplementary material for: Predicting COVID-19 cases with unknown homogeneous or heterogeneous resistance to infectivity
Source: PLoS One. 2021 Jul 15;16(7):e0254313. doi: 10.1371/journal.pone.0254313 (PMC8282037; doi:10.1371/journal.pone.0254313)
Supplement: S1 Appendix — (DOCX) [file pone.0254313.s002.docx]

**S1 Appendix**

A few explanations are worthwhile here for the sake of predicting the future COVID-19 cases. First, a non-intuitive explanation is that when the $jumpRate_{(\theta,\tau)}$ is smaller, the expected number of future COVID-19 cases is larger and that is why COVID-19 is mysterious. Next, let us now look at other intuitive explanations. When the number $m$ of family gatherings is greater, the expected number of future COVID-19 cases is greater. In other words, the $jumpRate_{(\theta,\tau)}$ of COVID-19 cases is a factor not only of the predicted number but also as the heterogeneity (that is, variance) level of the COVID-19 incidences. When the $jumpRate_{(\theta,\tau)}$ is larger, the expected number of future COVID-19 cases is closer to its variance, illustrating an equal variance scenario.

When the *heterogeneity resistant level*,$\tau$, to COVID-19 increases more and more from its baseline value.$\tau=1$ (which is indicative of best homogeneously immune level with respect to COVID-19), the variance,$Var(Y)$changes in proportion to the expected number,$E(Y)$of COVID-19 cases. The proportionality,$\frac{(1-\theta)^{(\tau-1)}}{\theta}$ reduces as the *heterogeneously resistant level*,$\tau$increases.

Note that Expressions (1) through (12) are displayed in the text. Expression numbers in this Appendix are in continuation.

When$\tau=1$, the future expected value and the variance do also change to

$E(Y)=\frac{m(1-\theta)}{\theta}=mOdds_{IB}^{safe}$ (13)

and

$Var(Y)=\frac{m(1-\theta)}{\theta^{2}}=\frac{E(Y)}{\theta}=E(Y)\{1+Odds_{IB}^{safe}\}$ (14)

With homogeneously immune participants in the family gathering, which is indicated by the traditional IB process, where$Odds_{IB}^{safe}=\frac{(1-\theta)}{\theta}$. The predictor must realize that the number *m* of unions could also increase proportionally the number of COVID-19 cases and consequently might inflate the heterogeneity (that is, more variance). Unlike the traditional IB situation which exhibits only *over* variance, the new IRRIB model in this article portrays additionally *equal* and *under* variance. That is, in the case of *under variance,* note that $(1-\theta)^{(\tau-1)}<\theta$ or equivalently $\{\tau>1+odds_{IB}^{safe}\}if\_attendees\_are\_heterogeneously$ resistant to COVID-19 virus.

This is obtained after taking the logarithm on both sides and applying the Taylor’s series expansion:

$\ln\theta=\ln\{1-(1-\theta)\}\approx-(1-\theta);0<\theta<1$. (14)

Notice that the odds of being safe from COVID-19 among those with homogeneously immune is: $Odds_{IB}^{safe}=\frac{(1-\theta)}{\theta}$. Otherwise (that is, when $\tau<1+Odds_{IB}^{safe}$), the IRRIB process portrays only *over variance.* When$\tau=1+Odds_{IB}^{safe}$, the IRRIB process echoes *equality of expected value and variance*, countering that the equality of expected value and variance is unique to the Poisson process [6]. The odds for a *safe* situation in the family gathering involving attendees having *heterogeneous resistance to COVID-19*) against contracting COVID-19 is:$Odds_{\tau\neq1}^{safe}=\frac{\Pr(Y=0)}{\Pr(Y\geq1)}=\left|\frac{(1-\theta_{\tau\neq1})^{(\tau-1)}}{\theta}-1\right|;0<\theta<\frac{1}{\tau}<1$ (15)

in comparison to the

$Odds_{\tau=1}^{safe}=[(1-\theta_{\tau=1})^{-m}-1]^{-1};0<\theta<1$ (16)

for *safe* in the family gathering involving attendees who are *homogeneously immune to COVID-19*).

The other statistical properties of the IRRIB process are the following. The probability generating function (that is,$E(z^{Y})$) is

$E(z^{Y})=\sum_{y=0}^{\infty} z^{y}\Pr(y)=\frac{\theta}{(1-\theta)^{(\tau-1)}(1-z)+z}$. (17)

The moment generating function (that is,$E(e^{tY})=\sum_{y=0}^{\infty} e^{ty}\Pr(y)$) is easily seen to be $\frac{\theta}{(1-\theta)^{(\tau-1)}(1-e^{t})+e^{t}}$. (18)

The skewness is a measure of asymmetry. The skewness of IRRIB process is

$Skewness=\frac{2(1-\theta)^{(\tau-1)}-\theta}{\sqrt{m\tau(1-\theta)^{(\tau-1)}\{(1-\theta)^{(\tau-1)}-\theta\}}}$. (19)

The kurtosis is a measure of the tail thickness. The excessive kurtosis in IRRIB process is

$ExcessKurtosis=\frac{6}{m\tau}+\frac{\theta^{2}}{\{(1-\theta)^{(\tau-1)}-\theta\}}$. (20)

The Fisher’s expected information,$I(\theta,\tau,m)$ quantifies what a randomly selected sample informs about the *infectivity/risk parameter*, $\theta$. That is,

$I(\theta,\tau,m)=-E\{\partial_{\theta\theta}^{2}\mathrm{lnPr}(y)\}=\frac{m\tau(1-\theta)^{3(1-\tau)}}{\theta^{2}\{(1-\theta)^{(1-\tau)}-\theta\}}$, (21)

where the notationis the second derivative with respect to the infectivity parameter,$\theta$. The survival function of the IRRIB process is then

$survivalFunction_{IRRIB}=\Pr(Y\geq p)=P(F_{(2p,2m\tau)df}\leq\frac{m\tau\{(1-\theta)^{(\tau-1)}-\theta\}}{\theta});0<\theta<\frac{1}{\tau}<1.$ (22)

The *hazard rate* is recognized as a force of mortality.

The hazard rate,$h(y)$ of the IRRIB process is

$hazardRate=\frac{\Gamma(m\tau+y)\{\frac{\theta}{(1-\theta)^{(\tau-1)}}\}^{y}\{1-\frac{\theta}{(1-\theta)^{(\tau-1)}}\}^{m\tau}}{y!\Gamma(m\tau)P(F_{(2[y+1],2m\tau)df}\leq\frac{m\tau\{(1-\theta)^{(\tau-1)}-\theta\}}{\theta})};0<\theta<\frac{1}{\tau}<1.$ (23)

The Markov chain is a way of capturing the *memory* in the chance mechanism. It is known that the traditional geometric process maintains no memory [6]. Its expected value is that$\Pr(Y\geq q\left|Y\geq p\right.)=\Pr(Y\geq q)$. With $\tau=1$, we note a memory relation that

$\Pr(Y\geq q\left|Y\geq p\right.)=\frac{\Pr(Y\geq p+q)}{\Pr(Y\geq p)}=\frac{\theta^{p+q}}{\theta^{p}}=\theta^{q}=\Pr(Y\geq q);0<\theta<1$ (24)

confirming that the traditional geometric process does not hold any memory. Does the IRRIB process deviates from such lack of memory? To answer this question, we first notice$\Pr(Y\geq q\left|Y\geq p\right.)\neq\Pr(Y\geq q)$. It suggests that there is a memory in the chance-oriented health mechanism which is portrayed by IRRIB process. The memory level is

$\begin{matrix} healthSystem'sMemory \\ =\frac{P(F_{(2[p+q],2m\tau)df}\leq\frac{m\tau\{(1-\theta)^{(\tau-1)}-\theta\}}{\theta})}{P(F_{(2p,2m\tau)df}\leq\frac{m\tau\{(1-\theta)^{(\tau-1)}-\theta\}}{\theta})}-P(F_{(2q,2m\tau)df}\leq\frac{m\tau\{(1-\theta)^{(\tau-1)}-\theta\}}{\theta});0<\theta<\frac{1}{\tau}<1. \end{matrix}$ (25)

When$\tau=1$, corresponding to the family gathering in which the attendees are homogeneously immune to COVID-19, the survival function, hazard rate, and the memory of the traditional IB process become, respectively,

$survivalFunction=S(p,\theta,\tau=1)=P(F_{(2p,2m)df}\leq\frac{m(1-\theta)}{\theta});0<\theta<1,$ (26)

and

$heathSystem'sMemory=\frac{P(F_{(2[p+q],2m)df}\leq\frac{m(1-\theta)}{\theta})}{P(F_{(2p,2m)df}\leq\frac{m(1-\theta)}{\theta})}-P(F_{(2q,2m)df}\leq\frac{m(1-\theta)}{\theta});0<\theta<1$ (27)

Next, we discuss the estimation of the parameters of the IRRIB process and obtain parameter estimates for IB process as particular cases.

Consider a random sample$y_{1},y_{2},y_{3},...,y_{n}$of size$n$from the IRRIB process. We resort to the maximum likelihood estimate (MLE) of the parameters because of its virtue. That is, the maximum likelihood estimate of a function of the parameters is the function of their MLEs. In the case of homogeneously immune to COVID-19, the MLE of the risk to contract COVID-19 is

$\hat{\theta}_{\tau=1,mle}=(\frac{\bar{y}}{s_{y}^{2}})$, if $s_{y}^{2}>\bar{y}$and $\hat{\theta}_{\tau=1,mle}=(\frac{s_{y}^{2}}{\bar{y}+s_{y}^{2}})$, if $s_{y}^{2}>\bar{y}$. (28)

In the case of heterogeneously resisting to COVID-19, the MLEs are

$\hat{\theta}_{\tau\neq1,mle}=(\frac{s_{y}^{2}}{s_{y}^{2}+\bar{y}})$ and consequently, $\hat{\tau}_{mle,\tau\neq1}\approx\frac{(\frac{s_{y}^{2}}{\bar{y}})}{\hat{\theta}}$ (29)

To perform the test of hypothesis on the significance of the MLE, we could consider Neyman’s$C(\alpha)$methodology [14]. Regression based test was developed in Neyman [14] to perform a composite hypothesis in dealing with a non-Gaussian random sample and it is recognized as$C(\alpha)$ test. An alternative but easier approach to test the null hypothesis $H_{o}:\tau=1$ against an alternative hypothesis $H_{1}:\tau\neq1$ is developed below. The null hypothesis is rejected when the test statistic,

$z=\left(\frac{\bar{y}^{2}}{\left|s_{y}^{2}-\bar{y}\right|}\right)\sqrt{n-1}$ (30)

is significantly larger and accept the alternative hypothesis. In other words, the probability for the null hypothesis$H_{o}:\tau=1$ to be the true statement is

$p-value=\Phi[\left(\{\frac{\bar{y}^{2}}{\left|s_{y}^{2}-\bar{y}\right|}\}-E\{\frac{\bar{y}^{2}}{\left|s_{y}^{2}-\bar{y}\right|}\left|H_{o}\right.\}_{\tau=1}\right)\left(\sqrt{\mathrm{var}\{\frac{\bar{y}^{2}}{\left|s_{y}^{2}-\bar{y}\right|}\left|H_{o}\right.\}_{\tau=1}}\right)^{-1}]$, (31)

where $\Phi(.)$ is the Gaussian cumulative function,

$E\{\frac{\bar{y}^{2}}{\left|s_{y}^{2}-\bar{y}\right|}\left|H_{o}\right.\}_{\tau=1}$ and $Var\{\frac{\bar{y}^{2}}{\left|s_{y}^{2}-\bar{y}\right|}\left|H_{o}\right.\}_{\tau=1}$ (32)

are the expected value and variance under the null hypothesis. The null hypothesis$H_{o}:\tau=1$ is synonymous with the statement that the random sample is drawn from the IB process (which is characterized by$s_{y}^{2}>\bar{y}$). Consequently, the significance level for the null hypothesis$H_{o}:\tau=1$is

$p-value\approx\Phi[\{\frac{\bar{y}^{2}}{\left|s_{y}^{2}-\bar{y}\right|}-E\{\frac{\bar{y}^{2}}{\left|s_{y}^{2}-\bar{y}\right|}\left|H_{o}\right.\}_{\tau=1}/\sqrt{Var\{\frac{\bar{y}^{2}}{\left|s_{y}^{2}-\bar{y}\right|}\left|H_{o}\right.\}_{\tau=1}}]$ (33)

The *(statistical) power* of accepting an alternative value $H_{a}:\tau=\tau^{*}$is $power\approx1-\Phi(\tau^{*})$, with$\alpha=\Pr(\mathrm{Re}ject\_H_{0}:\tau=1)=0.05$ and

$\tau^{*}=\frac{1.645\sqrt{\mathrm{var}\{\frac{\bar{y}^{2}}{\left|s_{y}^{2}-\bar{y}\right|}\left|H_{o}\right.\}_{\tau=1}}+E\{\frac{\bar{y}^{2}}{\left|s_{y}^{2}-\bar{y}\right|}\left|H_{o}\right.\}_{\tau=1}-E\{\frac{\bar{y}^{2}}{\left|s_{y}^{2}-\bar{y}\right|}\left|H_{1}\right.\}_{\tau\neq1}}{\sqrt{\mathrm{var}\{\frac{\bar{y}^{2}}{\left|s_{y}^{2}-\bar{y}\right|}\left|H_{o}\right.\}_{\tau=1}}}$ (34)

The hazard rate to contract COVID-19 in a situation in which the family gathering consists of attendees having *heterogeneous resistance to COVID-19* is

$$h(r\left|\theta,\tau)=1-\frac{S(r+1,\theta_{\tau\neq1},\tau)}{S(r,\theta_{\tau\neq1},\tau)}=1-\frac{P(F_{(2[r+1],2m\tau)df}\leq\frac{m\tau\{(1-\theta_{\tau\neq1})^{(\tau-1)}-\theta_{\tau\neq1}\}}{\theta})}{P(F_{(2r,2m\tau)df}\leq\frac{m\tau\{(1-\theta_{\tau\neq1})^{(\tau-1)}-\theta_{\tau\neq1}\}}{\theta_{\tau\neq1}})}\right.;0<\theta_{\tau\neq1}<\frac{1}{\tau}<1.$$

(35)

Initially (that is,$r=1$), the hazard rate is

$h(1\left|\theta_{\tau\neq1},\tau)\right.=1-\frac{P(F_{(4,2m\tau)df}\leq\frac{m\tau\{(1-\theta_{\tau\neq1})^{(\tau-1)}-\theta_{\tau\neq1}\}}{\theta_{\tau\neq1}})}{P(F_{(2,2m\tau)df}\leq\frac{m\tau\{(1-\theta_{\tau\neq1})^{(\tau-1)}-\theta_{\tau\neq1}\}}{\theta_{\tau\neq1}})};0<\theta<\frac{1}{\tau}<1$ (36)

in the family gathering’s attendees having *heterogeneous resistance to COVID-19* in comparison to

$h(1\left|\theta_{\tau=1},\tau=1)\right.=1-\frac{P(F_{(4,2m)df}\leq\frac{m(1-\theta_{\tau=1})}{\theta_{\tau=1}})}{P(F_{(2,2m)df}\leq\frac{m(1-\theta_{\tau=1})}{\theta_{\tau=1}})};0<\theta<1$ (37)

in a situation in which the family gathering consists of attendees having *homogeneously immune to COVID-19*. The reduction in the hazards due to having attendees with *heterogeneously resistant to COVID-19* in the family gathering is $\mathrm{Re}duction=h(1\left|\theta_{\tau=1},\tau=1)\right.-h(1\left|\theta_{\tau\neq1},\tau)\right.$.

We next consider a popular concept called *Tail Value at Risk* (TVaR) in the business world [15] and it is useful in the context of contracting COVID-19. That is,

$TVaR_{\tau}=E[Y\left|Y\geq1,\theta_{\tau\neq1},\tau\right.]\approx1+\frac{m\tau\{\frac{(1-\theta_{\tau\neq1})^{(\tau-1)}}{\theta_{\tau\neq1}}-1\}}{\theta_{\tau\neq1}P(F_{(2,2m\tau)df}\leq m\tau\{\frac{(1-\theta_{\tau\neq1})^{(\tau-1)}}{\theta_{\tau\neq1}}-1\})};0<\theta<\frac{1}{\tau}<1$ (38)

in the family gathering with attendees having *heterogeneous resistance to COVID-19* in comparison to

$TVaR_{\tau=1}=E[Y\left|Y\geq1,\theta_{\tau=1},\tau=1\right.]\approx1+\frac{m(1-\theta_{\tau=1})}{\theta_{\tau=1}P(F_{(2,2m)df}\leq\frac{m\{(1-\theta_{\tau=1})\}}{\theta_{\tau=1}})};0<\theta<1$ (39)

in the family gathering consisting of attendees having *homogeneous resistance to COVID-19*.

**Some mathematical derivations**

**A. Derivation of survival function**

$$\begin{matrix} survivalFunction_{IRRIB}=S(p,\theta,\tau)=\Pr(Y\geq p)=\sum_{i=p}^{\infty} \frac{\Gamma(m\tau+i)}{i!\Gamma(m\tau)}\{\frac{\theta}{(1-\theta)^{(\tau-1)}}\}^{i}\{1-\frac{\theta}{(1-\theta)^{(\tau-1)}}\}^{m\tau} \\ =P(F_{(2p,2m\tau)df}\leq\frac{m\tau\{(1-\theta)^{(\tau-1)}-\theta\}}{\theta});0<\theta<\frac{1}{\tau}<1. \end{matrix}$$

(40)

**B. Derivation of hazard rate**

$$\begin{matrix} hazardRate=h(y)=\frac{\Pr(y)}{S(y+1)} \\ =\frac{\Gamma(m\tau+y)\{\frac{\theta}{(1-\theta)^{(\tau-1)}}\}^{y}\{1-\frac{\theta}{(1-\theta)^{(\tau-1)}}\}^{m\tau}}{y!\Gamma(m\tau)P(F_{(2[y+1],2m\tau)df}\leq\frac{m\tau\{(1-\theta)^{(\tau-1)}-\theta\}}{\theta})};0<\theta<\frac{1}{\tau}<1. \end{matrix}$$

(41)

**C. Markov chain**

$$\Pr(Y\geq q\left|Y\geq p\right.)=\frac{\Pr(Y\geq p+q)}{\Pr(Y\geq p)}=\frac{P(F_{(2[p+q],2)df}\leq\frac{\{(1-\theta)^{(\tau-1)}-\theta\}}{\theta})}{P(F_{(2p,2)df}\leq\frac{\{(1-\theta)^{(\tau-1)}-\theta\}}{\theta})}\neq P(F_{(2q,2)df}\leq\frac{\{(1-\theta)^{(\tau-1)}-\theta\}}{\theta})$$

(42)

**D. Derivation of memory**

$$\begin{matrix} healthSystem'sMemory=\Pr(Y\geq q\left|Y\geq p\right.)-\Pr(Y\geq q) \\ =\frac{P(F_{(2[p+q],2m\tau)df}\leq\frac{m\tau\{(1-\theta)^{(\tau-1)}-\theta\}}{\theta})}{P(F_{(2p,2m\tau)df}\leq\frac{m\tau\{(1-\theta)^{(\tau-1)}-\theta\}}{\theta})}-P(F_{(2q,2m\tau)df}\leq\frac{m\tau\{(1-\theta)^{(\tau-1)}-\theta\}}{\theta});0<\theta<\frac{1}{\tau}<1. \end{matrix}$$

(43)

**E. Derivation of memory in union with homogeneous attendees**

$\begin{matrix} heathSystem'sMemory=\Pr(Y\geq q\left|Y\geq p\right.)-\Pr(Y\geq q) \\ =\frac{P(F_{(2[p+q],2m)df}\leq\frac{m(1-\theta)}{\theta})}{P(F_{(2p,2m)df}\leq\frac{m(1-\theta)}{\theta})}-P(F_{(2q,2m)df}\leq\frac{m(1-\theta)}{\theta});0<\theta<1 \end{matrix}$ (44)

**F. Derivation of maximum likelihood estimators for the parameters**

The log-likelihood function is

$$\begin{matrix} \ln L(y_{1},y_{2},y_{3},...,y_{n},m,\tau,\theta) \\ =\sum_{i=1}^{n} \{\ln\Gamma(m\tau+y_{i})-\ln y_{i}\}+n\bar{y}\{\ln\theta-(\tau-1)\ln(1-\theta)\}+n\{m\tau\ln(1-\frac{\theta}{(1-\theta)^{(\tau-1)}})-\ln\Gamma(m\tau)\}. \end{matrix}$$

(45)

Remember that the parameter$m$is assumed to be known. To get the MLE for the risk parameter,$\theta$and the restriction parameter,$\tau$, their score functions$\partial_{\theta}\ln L$and$\partial_{\tau}\ln L$are equated to zero simultaneously and solved. For this purpose, we need to solve the score function$\frac{\theta}{(1-\theta)^{(\tau-1)}}=\frac{\bar{y}}{s_{y}^{2}}$we take its logarithm and rewrite as below. That gives

$\ln[1-(1-\theta)]-(\tau-1)\ln(1-\theta)=\ln(\frac{\bar{y}}{s_{y}^{2}})$ (46)

Applying an approximation $-\ln(1-u)\approx u;0<u<1$ on both sides and simplifying, we note that

$-(1-\theta)+(\tau-1)\theta\approx\ln(\frac{\bar{y}}{s_{y}^{2}})$, which is equivalent to $\tau=\frac{\{1+\ln(\frac{\bar{y}}{s_{y}^{2}})\}}{\theta}$ . (47)

In the case of homogeneously immune to COVID-19, the MLE of the risk to contract COVID-19 is $\hat{\theta}_{\tau=1,mle}=(\frac{\bar{y}}{s_{y}^{2}})$, if $s_{y}^{2}>\bar{y}$and $\hat{\theta}_{\tau=1,mle}=(\frac{s_{y}^{2}}{\bar{y}+s_{y}^{2}})$, if $s_{y}^{2}>\bar{y}$. In the case of heterogeneously resisting to COVID-19, the MLEs are

$\hat{\theta}_{\tau\neq1,mle}=(\frac{s_{y}^{2}}{s_{y}^{2}+\bar{y}})$ and consequently, $\hat{\tau}_{mle,\tau\neq1}\approx\frac{(\frac{s_{y}^{2}}{\bar{y}})}{\hat{\theta}}$. (48)

**G. Derivation of general and special expressions**

We derive their expressions in a general framework and then deduct separately expressions under the null and alternative hypothesis. Recall what is already known that the sample expected value and variance are stochastically independent when the data are drawn from the Gaussian population (Stuart and Ord, 2015) but not necessarily so from the IRRIB process. Note that

$$E\{\frac{\bar{y}^{2}}{\left|s_{y}^{2}-\bar{y}\right|}\}=\frac{E(\bar{y}^{2})}{E(\left|s_{y}^{2}-\bar{y}\right|)}\{1+\frac{Var(\left|s_{y}^{2}-\bar{y}\right|)}{E(\left|s_{y}^{2}-\bar{y}\right|)}\}-\rho_{\bar{y}^{2},\left|s_{y}^{2}-\bar{y}\right|}\frac{\sqrt{Var(\bar{y}^{2})Var(\left|s_{y}^{2}-\bar{y}\right|)}}{\{E(\left|s_{y}^{2}-\bar{y}\right|)\}^{2}}$$

(49)

and

$$Var\{\frac{\bar{y}^{2}}{\left|s_{y}^{2}-\bar{y}\right|}\}=\{\frac{E(\bar{y}^{2})}{E(\left|s_{y}^{2}-\bar{y}\right|)}\}^{2}\{\frac{Var(\bar{y}^{2})}{[E(\bar{y}^{2})]^{2}}+\frac{Var(\left|s_{y}^{2}-\bar{y}\right|)}{[E(\left|s_{y}^{2}-\bar{y}\right|)]^{2}}\}-2\rho_{\bar{y}^{2},\left|s_{y}^{2}-\bar{y}\right|}\frac{\sqrt{Var(\bar{y}^{2})Var(\left|s_{y}^{2}-\bar{y}\right|)}}{\{E(\left|s_{y}^{2}-\bar{y}\right|)\}\{E(\bar{y}^{2})\}}.$$

(50)

See [16] for details for the expectation and variance of the correlated expressions in the ratio). Note that

$E(\bar{y}^{2})\approx\frac{m\tau}{n}\{\frac{(1-\theta)^{(\tau-1)}}{\theta}\}^{2};0<\theta<\frac{1}{\tau}<1$ (51)

$Var(\bar{y}^{2})\approx\frac{(m\tau)^{3}}{n}\{\frac{(1-\theta)^{(\tau-1)}}{\theta}\}^{4};0<\theta<\frac{1}{\tau}<1$, (52)

$E(\left|s_{y}^{2}-\bar{y}\right|)\approx(1-\frac{1}{n})m\tau\{\frac{(1-\theta)^{(\tau-1)}}{\theta}\}^{2};0<\theta<\frac{1}{\tau}<1$ (53)

and

$Var(\left|s_{y}^{2}-\bar{y}\right|)\approx\frac{m\tau}{n}\{\frac{(1-\theta)^{(\tau-1)}}{\theta}\}^{2};0<\theta<\frac{1}{\tau}<1$. (54)

Following an approach in Shanmugam (2020), the correlation,$\rho_{\bar{y}^{2},\left|s_{y}^{2}-\bar{y}\right|}$ between $\bar{y}^{2}$and$\left|s_{y}^{2}-\bar{y}\right|$is

$$\rho_{\bar{y}^{2},\left|s_{y}^{2}-\bar{y}\right|}=\frac{E(\left|s_{y}^{2}-\bar{y}\right|)}{E(\left|s_{y}^{2}-\bar{y}\right|)+E(\bar{y}^{2})}\approx\frac{\{\frac{\theta}{(1-\theta)^{(\tau-1)}}-(1-\frac{1}{n})\}}{\frac{\theta}{(1-\theta)^{(\tau-1)}}-(1-\frac{1}{n})+m\tau};0<\theta<\frac{1}{\tau}<1$$

(55)

and hence, from (14), we note that

$$E\{\frac{\bar{y}^{2}}{\left|s_{y}^{2}-\bar{y}\right|}\}\approx\frac{m\tau}{(n-1)}\{1-\frac{\rho_{\bar{y}^{2},\left|s_{y}^{2}-\bar{y}\right|}\theta}{(1-\theta)^{(\tau-1)}}\};0<\theta<\frac{1}{\tau}<1$$

(56)

and

$Var\{\frac{\bar{y}^{2}}{\left|s_{y}^{2}-\bar{y}\right|}\}\approx\frac{m\tau}{(n-1)}\{\frac{\theta}{(1-\theta)^{(\tau-1)}}\}\{1-\rho_{\bar{y}^{2},\left|s_{y}^{2}-\bar{y}\right|}\};0<\theta<\frac{1}{\tau}<1$.

(57)

To calculate the p-value, we need specific expressions for

$E\{\frac{\bar{y}^{2}}{\left|s_{y}^{2}-\bar{y}\right|}\left|H_{o}\right.\}_{\tau=1}$ (58)

and $Var\{\frac{\bar{y}^{2}}{\left|s_{y}^{2}-\bar{y}\right|}\left|H_{o}\right.\}_{\tau=1}$. (59)

To compute the statistical power of accepting the alternative hypothesis $H_{1}:\tau\neq1$ when it is true, we need expressions for

$E\{\frac{\bar{y}^{2}}{\left|s_{y}^{2}-\bar{y}\right|}\left|H_{1}\right.\}_{\tau\neq1}$ and $Var\{\frac{\bar{y}^{2}}{\left|s_{y}^{2}-\bar{y}\right|}\left|H_{1}\right.\}_{\tau\neq1}$. (60)

**References**

14. Neyman J. Optimal asymptotic tests of composite hypotheses. In Probability and Statistics: The Harold Cramer Volume. New York: Wiley, 1959.

15. Khokhlov V. Conditional value-at-risk for elliptical distributions, Evropský časopis Ekonomiky a Managementu 2016; 2 (6): 70–79.

16. Blumenfeld D. Operations Research Calculations Handbook. Boca Raton (FL): CRC Press; 2010.
